# Microbial Unmasking of Plant Glycosides

**DOI:** 10.1128/mBio.02433-17

**Published:** 2018-01-30

**Authors:** Kristen A. Biernat, Bo Li, Matthew R. Redinbo

**Affiliations:** a Department of Chemistry, University of North Carolina at Chapel Hill, Chapel Hill, North Carolina, USA; b Department of Microbiology, University of North Carolina at Chapel Hill, Chapel Hill, North Carolina, USA; c Integrative Program for Biological & Genome Sciences, University of North Carolina at Chapel Hill, Chapel Hill, North Carolina, USA; d Department of Biochemistry, University of North Carolina at Chapel Hill, Chapel Hill, North Carolina, USA

**Keywords:** enzymes, gut microbiota, plant glycosides

## Abstract

The therapeutic potential of plants is widely recognized and harnessed in plant-based remedies and drug discovery. However, the factors that modulate the bioavailability and bioactivities of plant-derived phytochemicals are poorly understood. In a recent article in *mBio*, M. C. Theilmann et al. (mBio 8:e01421-17, 2017, https://doi.org/10.1128/mBio.01421-17) describe how one gut microbe, *Lactobacillus acidophilus*, catalytically unwraps plant glucosides to make deglucosylated bioactive aglycones available to human tissues. They demonstrate that understanding the metabolism of plant glycosides by intestinal bacteria is essential to appreciating how bacteria manipulate the levels of bioactive plant metabolites in the human host.

## COMMENTARY

Humans have always leveraged the healing power of plants. The desire to maintain health and combat disease compelled ancient populations to observe and document the effects of edible resources in their environment. As this information was passed down through generations, civilizations acquired a thorough understanding of which plants possessed therapeutic potential and which were toxic or deadly. Plants believed to possess beneficial properties were incorporated into the medicinal practices of the earliest societies. The ancient Egyptians, for example, used willow tree leaves to relieve joint pain (1), and in the 4th century, the Chinese described using the sweet wormwood plant to treat the symptoms of malaria (2). Such knowledge then provided the foundation for pharmaceutical discovery and development. The same leaves, bark, and roots used in traditional medicinal practices became a source of our earliest drugs or drug leads. In the case of willow tree leaves, the glyosidic phytochemical salicin was identified as the compound responsible for pain relief and provided a template for the design of aspirin, one of the most commonly used drugs in the world today (1).

While plant-derived drugs continue to aid in the treatment and prevention of disease in the 21st century, alternative medicinal practices reminiscent of those of ancient times have also grown in popularity. People have embraced herbal supplements, functional food diets, probiotics, prebiotics, and “nutraceuticals” as a means of acquiring dietary compounds associated with anticancer, anti-inflammatory, and antioxidant activities (3, 4). Compared to the absorption, distribution, and potency of drugs, which have been intensively studied, the intake and levels of phytochemicals derived directly from dietary sources are less understood, more variable, and difficult to regulate (5). For instance, a complex milieu of dietary fiber must be metabolized by gastrointestinal (GI) tract bacteria to free up embedded phytochemicals, many of which also then require processing by gut microbes to their bioactive forms. Therefore, gaining a detailed understanding of how the gut microbiota modulates the bioactivities and bioavailability of phytochemicals is especially crucial in beginning to understand the efficacy of these biologically active plant-based remedies. The recent study by Theilmann et al. (6) contributes significantly to this underexplored area of research by providing insight into how specific GI bacteria interact with plant glycosides.

The bioavailability and bioactivities of plant glycosides, such as salicin, depend greatly on their conjugation to sugar moieties; plant glycosides are more polar and often less biologically active than the deconjugated aglycone. For this reason, microbes that utilize these plant glycosides as a source of energy are key players in regulating the levels of the bioactive aglycones available to human tissues (7). These bacteria possess transport proteins that import the plant glycoside into the cell and hydrolytic enzymes that cleave off the sugar moiety. The bioactive aglycone is then excreted and available for interactions with intestinal epithelial cells or other members of the gut microbiota. The specific bacteria and proteins involved in plant glycoside utilization have remained largely unknown, but Theilmann et al. (6) provide the first granularity about how *Lactobacillus acidophilus* interacts with these compounds.

Theilmann et al. (6) demonstrate that the probiotic strain *Lactobacillus acidophilus* NCFM grows on plant glycosides commonly found in the human diet, including the monoglucosylated compounds salicin and esculin and the diglucosylated compound amygdalin. Interestingly, they found that other common bacterial strains present in the GI tract did not grow on these phytoglucosides; this finding is important because it suggests that *L. acidophilus* occupies a unique metabolic niche for these compounds and uses them as carbon sources.

By analyzing the metabolites released by *L. acidophilus* into the culture supernatant, Theilmann et al. (6) confirm that *L. acidophilus* hydrolyzes the sugar moieties from these glucosides and exports the resultant aglycones out of the cell. These experiments highlight the potentially significant role that *L. acidophilus* plays in modulating the biological activities of specific phytochemicals. By metabolizing plant glucoside, *L. acidophilus* frees up aglycone for possible absorption and circulation in the host, increasing its bioavailability and, thus, its biological effect. This information provides insight into how the gut microbiota may contribute to various health effects in diet-based therapeutics. Further, this probiotic strain may be engineered or optimized to improve the bioavailability and activities of certain phytochemicals. Achieving this would realize the dream of partnering specific probiotic “enablers” with a plant-based dietary regimen designed to optimize one or more therapeutic outcomes.

In addition to linking *L. acidophilus* to the metabolism of plant-derived glucosides, Theilmann and colleagues (6) determined the specific microbial genes required for their processing. Using transcriptome sequencing (RNA-seq), they identified the gene loci in *L. acidophilus* that were upregulated upon growth on amygdalin, esculin, or salicin. One gene locus was highly expressed upon exposure to all three plant glucosides and contained the phosphotransferase (PTS) transporter LBA0725 and the phospho-β-glucosidase (P-Bgl) LBA0726. A second gene locus containing a related but distinct PTS, LBA0227, and a second type of the P-Bgl, LBA0225, was upregulated following growth on amygdalin but not on esculin or salicin. Theilmann et al. (6) validated the functionality of these two loci by analyzing the growth profiles of *L. acidophilus* deletion mutants. Based on their results, they attributed the cleavage of the monoglucosylated compounds esculin and salicin to the LBA0726 P-Bgl and the complete deglycosylation of the diglucosylated compound amygdalin to both glucosidases, with the LBA0225 P-Bgl recognizing the β-(1,6)-diglucoside moiety. Importantly, the authors’ data provide a means of predicting the metabolism of certain plant glycosides in Lactobacilli based on the presence or absence of related gene loci.

Can this predictive power be extended to account for structural differences in these related phospho-β-glucosidases? Subtle differences in active-site architectures might provide explanations for distinct plant glucoside processing specificities among closely related phospho-β-glucosidases.

**FIG 1:**
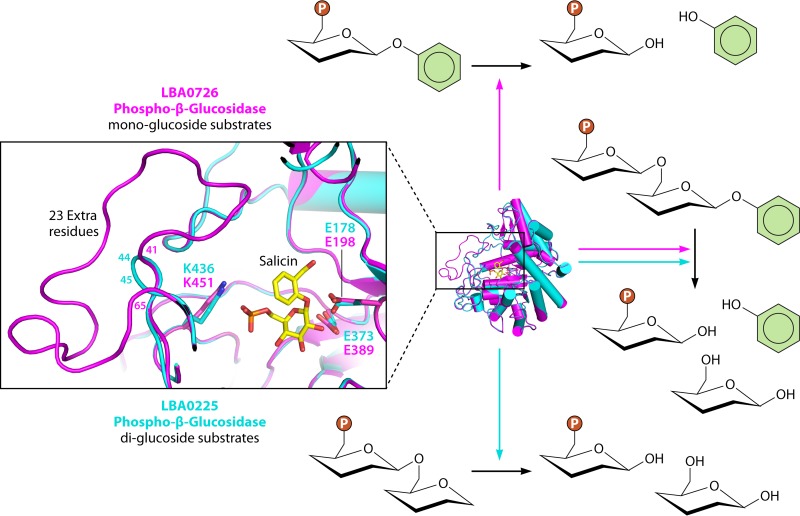
Superposition of the active sites of structural models of the phospho-β-glucosidases LBA0726 (magenta) and LBA0225 (cyan) from *Lactobacillus acidophilus* NCFM. LBA726 contains a much longer predicted active-site-adjacent loop (residues 41 to 65) than LBA0225 (residues 44 to 45), which may explain why the former processes small mono-glucoside substrates while the latter acts on di-glucosides. Salicin, a mono-glucoside, is docked to the active sites of these models. The lysine residues important for phosphate binding (K436, K451) and the two catalytic glutamic acids of each enzyme are annotated.

Thus, we modeled the structures of the two phospho-β-glucosidases LBA0726 and LBA0225 (8) identified by Theilmann et al. (6), which are upregulated in the presence of plant glucosides. We found that while they are predicted to have similar overall folds, the two proteins differ in the lengths of one loop near their respective active sites (Fig. 1). LBA0726, which was shown to process monoglucosides, contains a loop that is 23 amino acids longer than that of LBA0225, which processes diglucosides. Such structural distinctions may reflect differences in substrate specificity. Given that LBA0225 recognizes the β-(1,6)-diglucoside moiety, its shorter loop may permit more room in its active site to allow for larger substrates, like amygdalin, to bind. Likewise, the longer loop in LBA0726 may prevent amygdalin and other larger, diglucosylated compounds from entering its active site and instead may provide a more intimate space in which to align its monoglucosylated substrates, like salicin and esculin, for catalysis. Further work is necessary to confirm the role that such loops play in enzyme function, but these differences may provide another means of predicting the differential processing of plant glycosides.

In antiquity, humans had to rely on trial and error to determine the therapeutic properties of plant materials, and an error could have been deadly. Fortunately, our knowledge about the phytochemicals available in foods and other plants has significantly advanced. As demonstrated by Theilmann et al. (6), specific microbes can now be linked to the processing of phytoglycosides, which is important in understanding, and potentially manipulating, the levels of their bioactive metabolites. Further genetic and structural analyses may aid in predicting and optimizing the utilization of diverse phytochemicals. Indeed, in the future, we may see the dietary fiber that surrounds different plant-based foods as simply the delivery vehicle for the more precious phytochemical cargo carried within, a cargo that must be unpacked by the gut microbiota before their contents can be enjoyed by the grateful human host.
